# Long interspersed nuclear element-1 expression and retrotransposition in prostate cancer cells

**DOI:** 10.1186/s13100-017-0106-z

**Published:** 2018-01-03

**Authors:** Erica M. Briggs, Susan Ha, Paolo Mita, Gregory Brittingham, Ilaria Sciamanna, Corrado Spadafora, Susan K. Logan

**Affiliations:** 1 Department of Biochemistry and Molecular Pharmacology, New York University School of Medicine, New York, NY 10016 USA; 2 Department of Urology, New York University School of Medicine, New York, NY 10016 USA; 3 Institute for Systems Genetics, New York University School of Medicine, New York, NY 10016 USA; 4 National Health Institute, Rome, Italy; 5 Institute of Translational Pharmacology, National Research Council, Rome, Italy

**Keywords:** Transposable element, Prostate cancer, Tumor cell biology, Protein expression, Fluorescence, LINE-1, Retrotransposition

## Abstract

**Background:**

Long Interspersed Nuclear Element-1 (LINE-1) is an autonomous retrotransposon that generates new genomic insertions through the retrotransposition of a RNA intermediate. Expression of LINE-1 is tightly repressed in most somatic tissues to prevent DNA damage and ensure genomic integrity. However, the reactivation of LINE-1 has been documented in cancer and the role of LINE-1 protein expression and retrotransposition has become of interest in the development, progression, and adaptation of many epithelial neoplasms, including prostate cancer.

**Results:**

Here, we examined endogenous LINE-1 protein expression and localization in a panel of prostate cancer cells and observed a diverse range of LINE-1 expression patterns between cell lines. Subcellular localization of LINE-1 proteins, ORF1p and ORF2p, revealed distinct expression patterns. ORF1p, a nucleic acid chaperone that binds LINE-1 mRNA, was predominantly expressed in the cytoplasm, with minor localization in the nucleus. ORF2p, containing endonuclease and reverse transcriptase domains, exhibited punctate foci in the nucleus and also displayed co-localization with PCNA and γH2AX. Using a retrotransposition reporter assay, we found variations in LINE-1 retrotransposition between cell lines.

**Conclusions:**

Overall, our findings reveal new insight into the expression and retrotransposition of LINE-1 in prostate cancer. The prostate cancer cells we investigated provide a unique model for investigating endogenous LINE-1 activity and provide a functional model for studying LINE-1 mechanisms in prostate cancer.

**Electronic supplementary material:**

The online version of this article (10.1186/s13100-017-0106-z) contains supplementary material, which is available to authorized users.

## Background

Long Interspersed Nuclear Element-1 (LINE-1) is an autonomous, non-long terminal repeat retrotransposon that constitutes approximately 17% of the human genome [1]. Through the utilization of a RNA intermediate, LINE-1 creates new genomic insertions via a “copy and paste” mechanism known as retrotransposition. While ~500,000 copies of LINE-1 exist throughout the human genome, most are incapable of retrotransposition due to 5′ truncations, point mutations, or inversion; reducing full length, retrotransposition competent LINE-1 to 80-100 copies [2]. Full length LINE-1 mRNA consists of a 5’ UTR, containing an internal promoter, followed by two open reading frames coding for proteins ORF1p and ORF2p, and is terminated by a 3’UTR with a polyA sequence [3]. ORF1 protein (ORF1p) functions as a nucleic acid chaperone that binds LINE-1 mRNA in the cytoplasm during the retrotransposition cycle [4]. ORF2 protein (ORF2p) encodes the endonuclease and reverse transcriptase required for retrotransposition and is translated through an unconventional termination/reinitiation mechanism, limiting its expression relative to ORF1p [5–7]. Recent proteomic studies, using highly purified LINE-1 RNPs, have demonstrated a 1:27 ORF2p:ORF1p ratio using L1RP overexpression [8]. Due to its lower expression, ORF2p detection has been a challenge in the field until very recently. During the retrotransposition cycle, ORF1p and ORF2p bind LINE-1 mRNA in the cytoplasm, forming the ribonucleoprotein (RNP). The RNP is then transported from the cytoplasm to the nucleus through an unknown mechanism. Once in the nucleus, ORF2p creates a single stranded nick in the DNA through its endonuclease domain [6]. The reverse transcriptase domain of ORF2p then utilizes the LINE-1 mRNA as a template and creates a new LINE-1 insertion through Target Primed Reverse Transcription (TPRT) [9]. The retrotransposition cycle is thought to play a role in genomic variation and evolution, and more recently became of interest due to its role in disease initiation and progression [10].

Because of its ability to create new genomic insertions, LINE-1 expression is tightly repressed in most somatic tissues to ensure genomic stability. Mechanisms of LINE-1 repression include DNA methylation, histone modification, and RNA interference [11–13]. Yet, in many cancers, especially those of epithelial origin, reactivation of LINE-1 mRNA and protein expression have been observed [14–16]. Expression of LINE-1 ORF1p has been observed in 40-50% of prostate tumors, while ORF2p expression has recently been detected in both early and late stages of prostate cancer, yet, both proteins have been difficult to detect in matched normal prostate tissue compared to cancer [14, 17, 18]. Active LINE-1 retrotransposition provides a mechanism that could possibly drive cancer initiation and progression through genomic rearrangements, deletions, and translocations. Depending on the insertion site, LINE-1 can also affect gene expression of tumor suppressors or oncogenes, and has been proposed to potentially cause alternative splice variant formation [19–21]. Thus changes in LINE-1 activity are relevant to many cancers including prostate cancer. In a recent study, investigators analyzed 3′ transduction, an event wherein a unique sequence downstream of LINE-1 elements is co-transposed due to transcription past the repetitive LINE-1 sequence itself [22]. They found that across a variety of cancers, 53% had at least one 3′ transduction, indicative of a new retrotransposition. Interestingly, their analysis of a small number of metastatic prostate cancers suggested higher levels of retrotransposition in metastatic compared to primary prostate cancer.

Prostate cancer is one of the most commonly diagnosed malignancies in men and remains a leading cause of cancer related deaths [23]. The androgen steroid hormone receptor, a ligand-dependent transcription factor, is critical for growth and survival in both normal and malignant prostate cells and is the major therapeutic target in aggressive prostate cancer. Therapeutics for late stage prostate cancer, such as enzalutamide and abiraterone, target the activity of the androgen receptor by blocking androgen synthesis or androgen/AR binding [24, 25]. Yet, despite their initial efficacy, tumors often become resistant to therapy, and many patients progress to androgen independent, castration resistant prostate cancer (CRPC) [26]. Mechanisms of resistance include overexpression of the androgen receptor and formation of ligand independent androgen receptor splice variants; alterations that have been proposed consequences of LINE-1 retrotransposition [19, 27]. Alternatively, it has also been suggested that ORF1p may act as an AR coactivator in prostate cancer cells, driving growth and survival [28]. While evidence suggests that LINE-1 protein expression and retrotransposition may play a role in tumor initiation and progression, many questions remain regarding the role of LINE-1 in prostate cancer and the progression to castration resistant prostate cancer. To explore LINE-1 activity and expression in prostate cancer, we examined endogenous ORF1p and ORF2p expression and localization, as well as retrotransposition potential across a variety of prostate cancer cells.

## Methods

### Cell culture

E006AA-hT (CRL-3277), LNCaP (CRL-1740), PC3 (CRL-1435), DU-145 (HTB-81), VCaP (CRL-2876), and 22Rv1 (CRL-2505) cell lines were purchased from the ATCC. C4-2 cells were obtained from the Characterized Cell Line Core Facility at MD Anderson Cancer Center (Houston, TX). LAPC4, LNCaP-abl, and LNCaP-95 cell lines were generous gifts from R. Reiter, Z. Culig, and J. Isaacs, respectively. Cells were maintained as follows: LNCaP, 22Rv1 and C4-2 (RPMI 1640, 10% FBS), PC3 (Ham’s F-12 Nutrient Mixture, 10% FBS), LNCaP-95 and LNCaP-abl (RPMI 1640, phenol red free, 10% charcoal dextran stripped FBS), DU-145, VCaP, E006AAhT (DMEM, 10% FBS), and LAPC4 (Iscove’s DMEM, 10% FBS). Cells are routinely screened for mycoplasma.

### Retrotransposition assay

Cells were seeded in 6 cm plates and transfected with the synthetic human LINE-1 (pCEP-CMV-ORFeus-Hs-Puromycin-EGFPai (pPM016)) retrotransposition vector, pCEP-Puromycin-CMV-EGFP control (pLD107), or pCEP-Puromycin empty vector (pLD207) [8] using Lipofectamine 3000 (Life Technologies) according to the manufacturer’s instructions. Cells were then selected for 5 days with puromycin: LNCaP and 22RV1 (1 μg/mL), LAPC4 (2 μg/mL), and PC3 (0.5 μg/mL). GFP expression was quantified using a BD FACSCalibur flow cytometer using CellQuest Pro software and results analyzed using FlowJo 10.2 software [29, 30]. The vector only, negative control assessed the background fluorescence of each cell line and a cut off was established to exclude background fluorescence. The vector only samples had zero counts of GFP using this threshold and only fluorescence greater than this threshold was considered positive. pCEP-Puro-EGFP served as a positive control for EGFP expression, selection efficiency, and maintenance of the plasmids.

### Immunohistochemistry

LNCaP-abl xenografts were grown subcutaneously in the flank of NU/NU mice and harvested after 3.5 weeks as previously shown [31]. Immunohistochemistry was performed as described [32], using antibodies against ORF1 [14] and ORF2 [17]. 3,3′-Diaminobenzidine (DAB) was used as the chromogen to indicate positive reactivity against the antibody.

### Subcellular fractionation and western blot analysis

For each cell line, 10-15 × 10^6^ cells were used to isolate cellular fractions. Cellular fractionations were performed using a Subcellular Fractionation kit (Thermo Scientific) according to the manufacturer’s protocol with the addition of sodium orthovanadate. Total protein lysates were collected through lysis in RIPA buffer (50 mM Tris pH 8, 150 mM NaCl, 1% NP-40, 0.1% SDS, and 10 mM EDTA) supplemented with 10 μg/mL aprotonin and leuptin, and 0.1 mM PMSF and sodium orthovanadate, and protein was quantified using a Bradford assay. Lysates were resolved by SDS-PAGE on separate gels and probed with anti-ORF1 [14], anti-ORF2 [17], or anti-AR (Santa Cruz 441- sc7305). 25 μg or 100 μg of protein was loaded on gels to probe for ORF1p or ORF2p, respectively. Blots were stripped with Restore Western Blot Stripping Buffer (Thermo Scientific), cut, and re-probed for loading controls, anti-tubulin (Covance MMS-489P), anti-SP1 (Thermo Scientific PA5-29165) and anti-H3 (Abcam-1791). Quantification of ORF1p and ORF2p protein expression were performed using ImageJ 1.50i software. The densitometry of each band was calculated and the relative expression of the proteins normalized to tubulin.

### Immunofluorescence

Cells were plated on fibronectin coated glass coverslips and cultured overnight. Cells were either fixed in ice cold methanol (ORF2) or 10% neutral buffered formalin (ORF1) and permeabilized with 0.2% Triton-X-100 in PBS for 20 min. Slides were blocked with 5% BSA in TBS for 1 h and incubated with ORF1 or ORF2 antibodies overnight at 4 °C or 1 h at 37 °C, respectively. For double immunofluorescence, cells were incubated with ORF2 and γH2AX (Abcam-11,174) or PCNA (Abcam-18,197) antibodies overnight at 4 °C. Cells were washed with 0.1% Triton-X-100 in PBS and incubated with secondary antibodies, Alexa Fluor-555 or Alexa Fluor-647 (Thermo Fisher), in 5% normal goat and horse serum for 1 h at room temperature. After washing with 0.1% Triton-X-100 in PBS, cells were counterstained with DAPI mounting media (Vector Laboratories). ORF1p expression was visualized using an EVOS florescent microscope and imaged using an EVOS FL Auto Imaging System.

ORF2p expression and ORF2 co-localization was visualized using an Andor Yokogawa CSU-x spinning disc on a Nikon TI Eclipse confocal microscope and was recorded with an scMOS (Prime95B, Photometrics) camera with a 20× objective (pixel size 0.48 μm). Images were acquired using Nikon Elements software and analyzed using ImageJ/Fiji [33]. To quantify the number of cells that contained at least one co-localized foci, we first identified the total number of cells present in a 20× frame by counting DAPI positive nuclei. Next, we counted all cells with at least one co-localization foci, evident by the yellow signal from overlapping ORF2p foci (red) and PCNA or γ-H2AX foci (green). The average number of cells with at least one co-localization signal and standard deviations were calculated.

## Results

### Prostate cancer cell lines express LINE-1 ORF1 and ORF2 protein

**Fig. 1:**
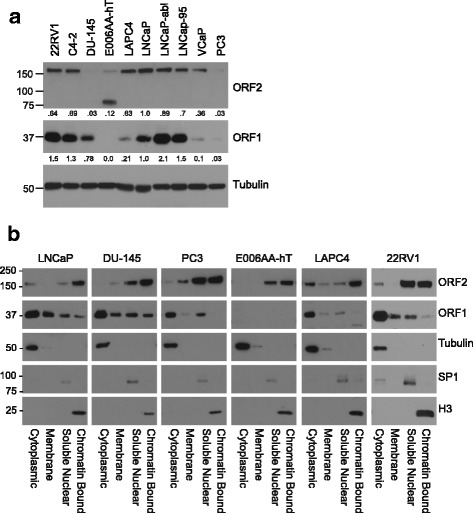
Endogenous ORF1p and ORF2p in prostate cancer cells. **a** Western blot analysis of total protein lysates from the indicated prostate cancer cell lines using antibodies against LINE-1 proteins, ORF1 or ORF2, and tubulin. The numbers below the panels denote that densitometry of the band relative to tubulin. **b** Subcellular fractions (cytoplasmic, membrane, soluble nuclear, and chromatin bound) of LNCaP, DU-145, PC3, E006AA-hT, LAPC4, and 22RV1 prostate cancer cell lines were subjected to western blot analysis. The indicated antibodies were used. Controls for the fractionation include tubulin (cytoplasm), SP1 (soluble nuclear) and histone H3 (chromatin)

To investigate endogenous LINE-1 proteins in prostate cancer we examined their expression in a variety of prostate cancer cells. We performed western blot analysis on total protein lysates from a panel of human prostate cancer cell lines using antibody against ORF1p [14]. We also utilized antibody against ORF2p [17] to determine whether ORF2 expression varied among the cell lines and whether ORF2p expression correlated with ORF1p levels (Fig. 1a). We observed that ORF1p is widely and differentially expressed among prostate cancer cell lines (Fig. 1a). We predicted that ORF2p would correlate with ORF1p since the two proteins are translated from a single bicistronic mRNA. Indeed, expression of ORF2p loosely correlated with ORF1p in the sense that most cell lines with higher levels of ORF1p also expressed at least some ORF2p. Consistent with a previous study [17], we find that AR positive LNCaP cells express ORF2p. The variation of ORF1p and ORF2p expression in the prostate cell lines did not correlate with common features of prostate cancer such as PTEN or p53 mutation, or the presence of the TMPRSS2/ERG gene fusion or AR splice variants (Additional file 1: Figure S1A and Additional file 2: Table S1). However, we note that among the AR negative cells, PC3 cells showed little ORF1p and ORF2p, and DU145 cells had low levels of ORF2p. We also examined expression in a cell line derived from an African American man (E006AA-hT) [34] since African Americans with prostate cancer have worse outcomes than other ethnic groups. Interestingly, E006AA-hT cells exhibited atypical LINE-1 protein expression, compared to the other prostate cell lines, where we detected ORF2p and no evident ORF1p. It is possible that the actively expressed LINE-1 loci in this cell line contain mutations or deletions in ORF1, limiting its expression/detection, but permitting the expression of ORF2p, as has been previously reported [35]. Additionally, western blot of total protein lysate of E005AA-hT had a significant band ~75KDa, unlike all other cell lines tested (Fig. 1a and Additional file 1: Figure S1B). It is possible this band represents a truncated form of ORF2p or a non-specific background of similar weight [36]. The predominant ORF2p band in all other cell lines was ~150KDa, the molecular weight of full length ORF2p.

### Subcellular localization of endogenous ORF1p and ORF2p in prostate cancer cells

To further investigate the expression patterns of LINE-1 proteins in prostate cancer cells, we conducted cell fractionation studies. As expected based on the literature we found that ORF1p was predominantly cytoplasmic (Fig. 1b) where it is known to form trimers and coat LINE-1 mRNA [37]. However, we could also detect low levels of ORF1p in the soluble nuclear and chromatin compartments, consistent with previous observations in exogenously expressed ORF1p and the fact that ORF1p is required, along with ORF2p, for LINE-1 retrotransposition [38, 39]. We observed that ORF2p was predominantly nuclear, localization that is necessary for its role in the retrotransposition cycle. Yet, we also detected lower levels of ORF2p in the cytoplasm where the RNP is formed. In contrast to ORF2p expression in total protein lysates, ORF2p was readily detectable across all the cell lines examined (Fig. 1b). The E006AA-hAT cells again did not have detectable ORF1p and the putative 75 kDa truncated ORF2p band (~75 kDa) was not the predominant band in the nuclear fractions (Fig. 1b). We note that there is a minor band at ~45 kDa in the nuclear fractions of DU-145, PC3, and E006AA-hT in western blots for ORF2 that may also represent a truncation (Additional file 3: Figure S2) [40].

**Fig. 2:**
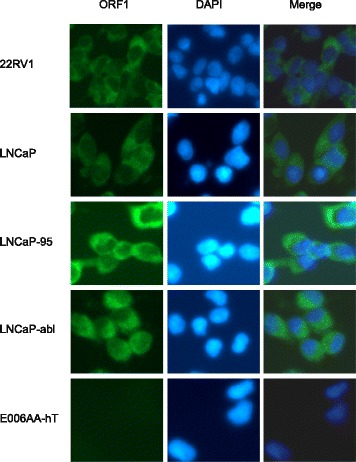
Immunofluorescence of endogenous ORF1p in a panel of prostate cancer cell lines. 22RV1, LNCaP, LNCaP-95, LNCaP-abl, and E006AA-hT cells were probed for endogenous ORF1p (green) and counterstained with DAPI (blue) to visualize nuclei. Representative epifluorescent images were taken under 20× magnification

**Fig. 3:**
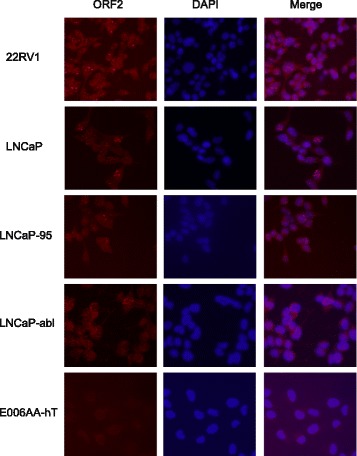
Immunofluorescence of endogenous ORF2p in a panel of prostate cancer cell lines. Cell lines, as described in Fig. 2, were probed for endogenous ORF2p (red) and counterstained with DAPI (blue) to visualize nuclei. Images were taken on a Nikon TI Eclipse confocal microscope under 20× magnification and a representative maximum-intensity projection is shown

As an additional method to examine the cellular compartments where ORF1p and ORF2p are present and to observe their pattern of localization across prostate cancer cell lines with varying dependence on androgens (Additional file 2: Table S1), we conducted immunofluorescence to detect endogenous proteins. We observed that ORF1p was predominantly cytoplasmic (Fig. 2) and detectable in the cell lines where we detected ORF1p by western blot (Fig. 1a). ORF1p was not detectable by immunofluorescence in E006AA-hT cells where we did not detect protein by western blot analysis. We also compared endogenous ORF2p localization across prostate cancer cell lines. In agreement with western blot analysis shown in Fig. 1b, ORF2p was primarily localized to nuclear foci (Fig. 3). Previously, De Luca et al. had demonstrated similar findings in melanoma A375 cells, where ORF2p and ORF1p formed distinct foci in the nucleus and cytoplasm [17]. ORF2p was not detectable in E006AA-hT cells by immunofluorescence, most likely due to diffuse expression or being below the level of detection, similar to western blots using total protein lysates compared to highly concentrated nuclear lysates.

**Fig. 4:**
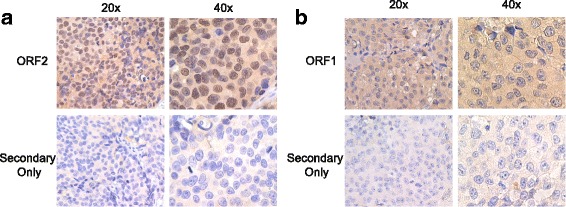
Endogenous LINE-1 expression in LNCaP-abl xenografts. Immunohistochemistry on LNCaP-abl xenograft tissue was performed. Sections were incubated with ORF2 (**a**) or ORF1 (**b**) antibody with positive reactivity indicated by brown staining (DAB). Sections were counterstained with hematoxylin (blue) to visualize nuclei. The secondary only, negative control is also shown. Representative pictures at 20× and 40× magnification are shown

To investigate ORF1p and ORF2p localization in an in vivo model of prostate cancer, we also performed immunohistochemistry on tissue sections from LNCaP-abl xenografts (Fig. 4). Our immunohistochemistry showed predominant cytoplasmic ORF1p localization and nuclear ORF2p localization, reiterating our findings in cultured cells.

### Retrotransposition in prostate cancer cells

**Fig. 5:**
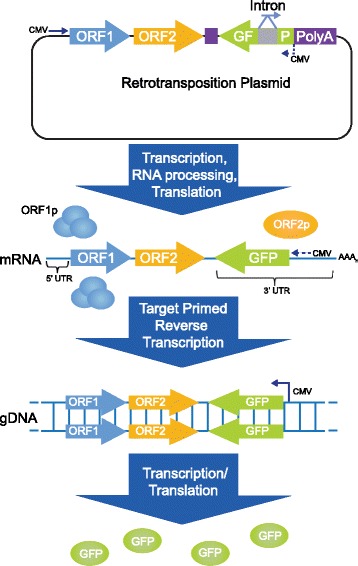
Retrotransposition Assay. The retrotransposition plasmid (pPM016) was transfected into cells. LINE-1 mRNA is expressed and processed, removing the γ globin intron from GFP during RNA splicing. ORF1p and ORF2p are translated and bind to LINE-1 mRNA, forming ribonucleoprotein (RNP) particles. The RNP enters the nucleus, where LINE-1 mRNA is reverse transcribed into host DNA by Target Primed Reverse Transcription (TPRT). After retrotransposition, cells expressing functional GFP are quantified by flow cytometry

The fact that we could detect ORF1p and ORF2p in nuclear fractions of prostate cancer cells (Fig. 1b) suggests that these cells are permissive for retrotransposition. Therefore, to determine if active retrotransposition could occur in these prostate cancer cells we conducted retrotransposition assays using a GFP-based retrotransposition reporter (Fig. 5). LNCaP, 22RV1, LAPC4, and PC3 cells were transfected with the retrotransposition reporter cassette containing a synthetic, recoded LINE-1 (ORFeus-Hs) under control of a CMV promoter. In these assays, the 3’ UTR region of LINE-1 contains an anti-sense GFP interrupted by a γ-globin intron in the opposite orientation of GFP. Functional GFP is only expressed after retrotransposition [8, 29, 30]. Consistent with the presence of ORF1p and ORF2p in the nucleus, retrotransposition occurs in LNCaP cells, 22Rv1, LAPC4, and PC3 cells. We observed that the highest retrotransposition frequency occurs in LNCaP cells (8.92%) and the lowest in LAPC4 cells (0.038%) (Table 1). The retrotransposition ability of the cells did not correlate to the endogenous expression level of ORF1p or ORF2p. It has been previously shown that increased transcription and expression of LINE-1 proteins using retrotransposition constructs do not necessarily correlate to retrotransposition frequency or endogenous levels of ORF1p and ORF2p [30, 41, 42].

**Table 1:** Retrotransposition frequency in prostate cell lines

Cell line	Retrotransposition frequency^a^
LNCaP	8.92 ± 0.04
22RV1	2.87 ± 0.16
LAPC4	0.38 ± 0.06
PC3	5.85 ± 0.61

^a^Percentage of EGFP positives cells. pCEP-CMV-ORFeus-Hs-Puro-GFPai (pPM016) was transfected into the above cell lines and EGFP expression quantified. The frequency is the mean of three independent experiments and error is represented by standard deviation

### ORF2 co-localizes with PCNA and γH2AX in a subset of prostate cancer cells

**Fig. 6:**
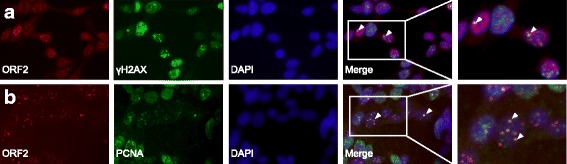
Co-localization of ORF2p and γH2AX (**a**) or PCNA (**b**) in LNCaP cells. Double immunofluorescence was performed using antibodies against ORF2 (red) and γH2AX (green) or PCNA (green). Cells were counterstained with DAPI. Images were taken on a Nikon TI Eclipse confocal microscope under 20× magnification and a representative maximum-intensity projection is shown. Arrowheads point to overlap of γH2AX and ORF2 (**a**) or PCNA and ORF2 (**b**) in the merged images

A recent study showed that ORF2 interacts with PCNA, a DNA processivity factor for DNA polymerases during DNA damage and repair [8]. ORF2 interacts with PCNA via a PCNA-interacting protein (PIP) box located between the ORF2 endonuclease and reverse transcriptase domains. Mutation of the PIP box disrupts ORF2:PCNA interaction and inhibits retrotransposition in reporter gene assays [8]. Mita et al. recently showed ORF2p and PCNA co-localization with exogenous LINE-1 expression [43]. Since these experiments were performed with overexpressed LINE-1 proteins, we wanted to determine if endogenous ORF2p and PCNA co-localized. We also examined subcellular localization of ORF2p with the marker of DNA damage, γH2AX since it has been shown that γH2AX and DNA damage increased upon LINE-1 upregulation [44, 45]. Double immunofluorescence was performed in LNCaP cells: ORF2p, and PCNA or γH2AX. We observed nuclear foci with detectable ORF2p, PCNA, or γH2AX (Fig. 6). Although not all ORF2p co-localizes with PCNA or γH2AX, 33.6 ± 4.3% of nuclei showed at least one foci of ORF2/PCNA co-localization, and 16.0 ± 5.8% of nuclei had at least one foci of ORF2/γH2AX co-localization (Fig. 6, arrowheads). The significance of ORF2p co-localization with γH2AX or PCNA and whether this can be altered by the phase of the cell cycle or by a variety of cell stresses is an important question for future investigation.

## Discussion

The de-repression of LINE-1 in cancer cells evokes many questions on the role of LINE-1 protein expression and retrotransposition in cancer progression. Many studies designed to investigate the mechanism of LINE-1 retrotransposition have relied on exogenous expression due to LINE-1 being normally repressed and the difficulty in detecting endogenous ORF2p. In this study, we utilized a newly developed ORF2p antibody and a well-established ORF1p antibody to investigate endogenous LINE-1 expression and localization in prostate cancer [14, 17]. Examination of ORF1p and ORF2p expression by western blot reveals a diverse range of expression patterns among prostate cancer cell lines. The variety of ORF1:ORF2 expression patterns suggest that there may be different mechanisms of LINE-1 regulation in different cell lines such as altered translation or degradation of LINE-1 proteins. In addition, LINE-1 is subject to transcriptional repression by epigenetic mechanisms including DNA methylation and histone modification, and these processes may also differ among the cell lines [46]. Previous studies have also demonstrated regulation of LINE-1 mRNA by MOV10, a RNA helicase, and RNA interference [47–51]. Thus, prostate cancer cell lines express a range of endogenous LINE-1 proteins that can be used to further elucidate the mechanism of LINE-1 regulation, as well as their possible role in cancer progression.

Exogenous expression of ORF1p is predominantly cytoplasmic and is also present in stress granules [52, 53]. Consistent with these findings, our results show diffuse cytoplasmic expression of ORF1p, with low levels in the nucleus. There are also some small punctate spots of ORF1p expression in the cytoplasm, which may be small stress granule formation but appear smaller than typical stress granules. We speculate that the robust expression of ORF1p in prostate cells will enable investigation into the impact of ORF1p on cancer progression.

ORF2p needs to be transported into the nucleus in order for retrotransposition to occur. While translocation of exogenous ORF2p to the nucleus has been observed, localization of endogenous ORF2p has not been demonstrated in prostate cancer cells. We found subcellular fractionation optimal for detecting low levels of ORF2p expression compared to total protein lysates, where we observed prominent full length bands in the soluble nuclear and chromatin bound fractions across multiple cell lines. Furthermore, our results show multiple clear punctate foci of ORF2p in the nuclei. In addition, immunofluorescence also revealed a population of ORF2p co-localized with PCNA or γH2AX and we speculate that these may be cells permissive to active retrotransposition. We also speculate that co-localization of ORF2p and γH2AX may indicate endonuclease independent retrotransposition, as previously observed [54]. Interestingly, while we observe ORF2 expression in most LNCaP cells, retrotransposition only occurs in approximately 9% of the cells. Retrotransposition frequency was also low in prostate tumors, where new LINE-1 insertions were identified though sequencing [22]. The localization patterns of ORF1p and ORF2p in the prostate cancer cells suggest that many prostate cancer cell lines are retrotransposition competent, a finding confirmed using retrotransposition assays. Further, we observed differential retrotransposition capability between cell lines. Such differences may reflect altered expression of viral host restriction pathway proteins that have been shown to inhibit retrotransposition [10, 55, 56]. Interestingly, whole exome sequencing data from LNCaP, 22RV1, and PC3 cells, available on cBioPortal (hosted by Center for Molecular Oncology at Memorial Sloan Kettering Cancer Center), have shown putative homozygous deletions of APOBEC3A and APOBEC3B in 22RV1 cells. APOBEC3C mRNA is also downregulated compared to reference populations in 22RV1 and LNCaP (z-scores −2.05 and −1.95, respectively) [57, 58].

The range of LINE-1 expression and retrotransposition in prostate cancer cell lines makes them an optimal model for investigating LINE-1 activity and repression in cancer, as well as a valuable tool to study the function of LINE-1 in cancer progression.

## Conclusions

In summary, we characterized endogenous LINE-1 expression and localization of 10 commonly used prostate cancer cell lines. We found varying levels of ORF1 and ORF2 expression levels between cell lines, representing a diverse set of prostate cancers. Cell lines with endogenous LINE-1 protein exhibited significant differences in retrotransposition activity. We also showed that expression patterns of endogenous LINE-1 proteins confirmed previous LINE-1 behavior, such as ORF2 co-localization either with PCNA and γH2AX. Our investigations have revealed a functional model for investigating endogenous LINE-1 activity in prostate cancer.

## Additional files

Additional file 1: Figure S1. Androgen receptor expression and full length of blots shown in Fig. 1a (.pdf) A) Western blot analysis of the androgen receptor (AR) in prostate cancer cell lines. Antibody against the AR recognized both full length (FL) and the V7 spliced variant. Tubulin was used as a loading control. B) High and low molecular weight regions surrounding ORF2 and ORF1. Overexposed images are from the same blot as shown in Fig. 1a. (PDF 2780 kb)

Additional file 2: Table S1. Molecular characteristics and origins of prostate cancer cell lines. (DOCX 43 kb)

Additional file 3: Figure S2. High and low molecular weight regions surrounding ORF1 and ORF2 western blots. (.pdf) Entire length of western blots presented in Fig. 1b, showing ORF2 and ORF1 expression in cellular fractions. (PDF 329 kb)

## Abbreviations

LINE-1: Long interspersed nuclear element-1
MOV10: Moloney leukemia virus 10
TPRT: Target primed reverse transcription
