# Supplementary figures and images for: Long interspersed nuclear element-1 expression and retrotransposition in prostate cancer cells
Source: Mob DNA. 2018 Jan 3;9:1. doi: 10.1186/s13100-017-0106-z (PMC5753491; doi:10.1186/s13100-017-0106-z)

A

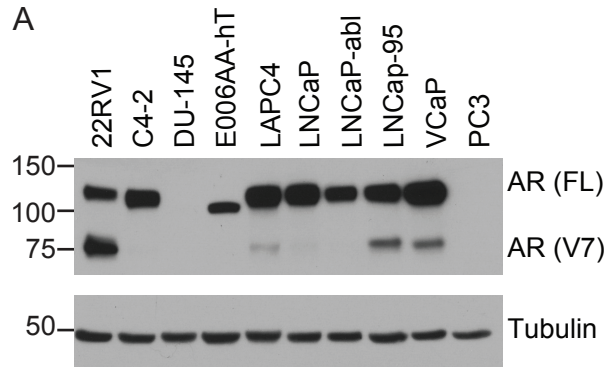

B

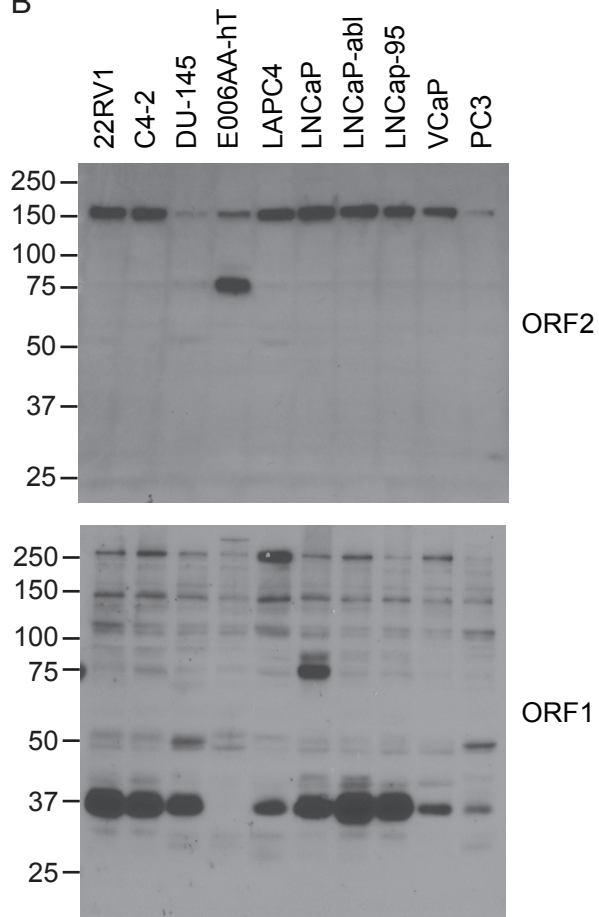

Supplement: Supplementary file 1 — Androgen receptor expression and full length of blots shown in Fig. 1a (.pdf) A) Western blot analysis of the androgen receptor (AR) in prostate cancer cell lines. Antibody against the AR recognized both full length (FL) and the V7 spliced variant. Tubulin was used as a loading control. B) High and low molecular weight regions surrounding ORF2 and ORF1. Overexposed images are from the same blot as shown in Fig. 1a. (PDF 2780 kb) [file 13100_2017_106_MOESM1_ESM.pdf]

ORF2 Long Exposure

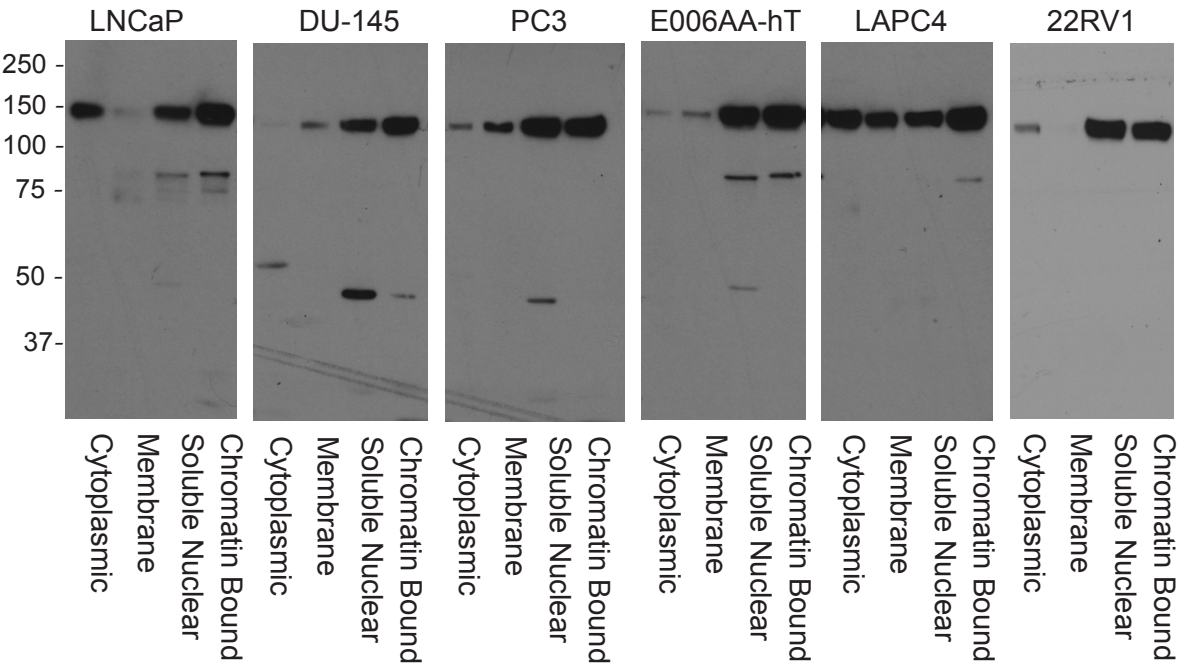

ORF1 Long Exposure

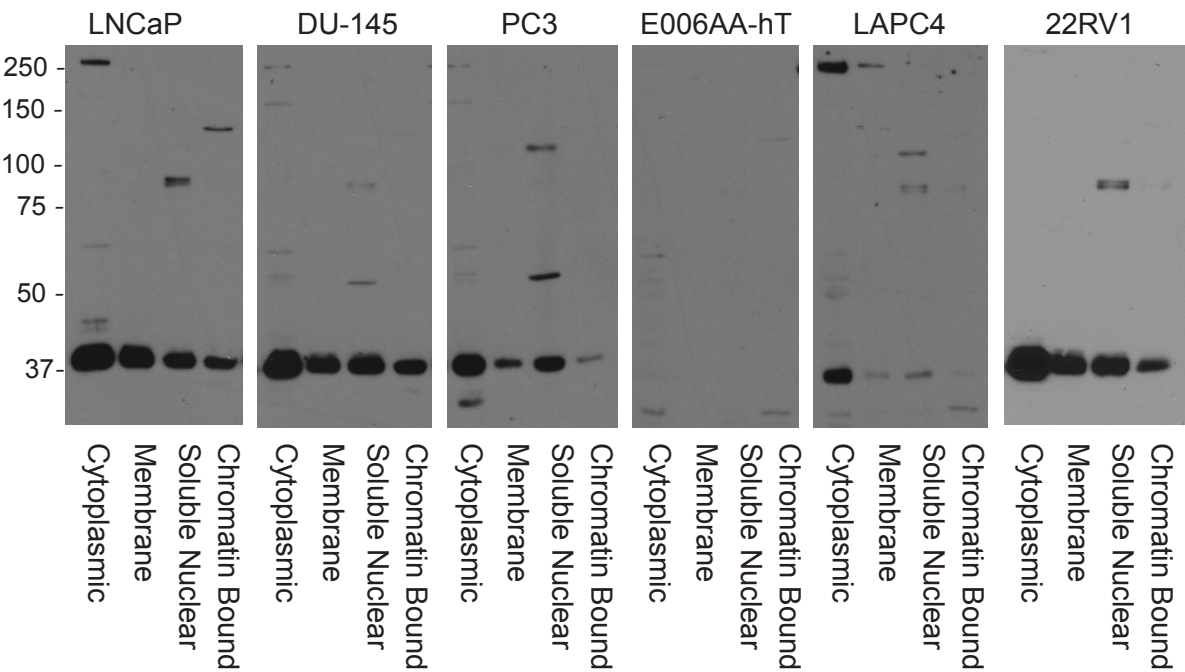

Supplement: Supplementary file 3 — High and low molecular weight regions surrounding ORF1 and ORF2 western blots. (.pdf) Entire length of western blots presented in Fig. 1b, showing ORF2 and ORF1 expression in cellular fractions. (PDF 329 kb) [file 13100_2017_106_MOESM3_ESM.pdf]
