# Supplementary material for: Long interspersed nuclear element-1 expression and retrotransposition in prostate cancer cells
Source: Mob DNA. 2018 Jan 3;9:1. doi: 10.1186/s13100-017-0106-z (PMC5753491; doi:10.1186/s13100-017-0106-z)
Supplement: Supplementary file 2 — Molecular characteristics and origins of prostate cancer cell lines. (DOCX 43 kb) [file 13100_2017_106_MOESM2_ESM.docx]

|  | Androgen Dependence | AR Status | AR Splice Variants | PTEN | p53 | Source | References |
| --- | --- | --- | --- | --- | --- | --- | --- |
| LNCaP | + | + |  | Inactivation | WT | Lymph node metastasis | [1-4] |
| LNCaP-95 | - | + | + |  |  | LNCaP cells cultured in androgen depleted media | [5] |
| LNCaP-abl | - | + |  |  |  | LNCaP cells cultured in androgen depleted media | [6] |
| C4-2 | - | + |  |  |  | LNCaP subline derived from castrated mice | [7] |
| 22RV1 | - | + | + | WT | Q331R | CWR22 (prostate tumor xenograft) subline derived from castrated mice | [8-10] |
| VCaP | + | + | + | Intact | A248W | Vertebral metastasis | [10-12] |
| LAPC4 | + | + | + ^a^ | WT | A175H | Lymph node metastasis | [12-14] |
| PC3 | - | − | - | Deficient | Mutations in codons 138 &169 | Lumbar vertebral metastasis | [4, 10, 15] |
| E006AA-hT | - | + |  | No Expression | High p53 expression | Primary Prostate tumor | [16] |
| DU-145 | - | - | - |  | P223L, V274F | Brain Metastasis | [4, 17, 18] |

**Additional File 2, Table S1.** Molecular characteristics and origin of prostate cancer cell lines.

^a^ our unpublished observation

1. Horoszewicz, J.S., et al., *The LNCaP cell line--a new model for studies on human prostatic carcinoma.* Prog Clin Biol Res, 1980. **37**: p. 115-32.

2. Sobel, R.E. and M.D. Sadar, *Cell lines used in prostate cancer research: a compendium of old and new lines--part 1.* J Urol, 2005. **173**(2): p. 342-59.

3. Vlietstra, R.J., et al., *Frequent inactivation of PTEN in prostate cancer cell lines and xenografts.* Cancer Res, 1998. **58**(13): p. 2720-3.

4. Carroll, A.G., et al., *p53 oncogene mutations in three human prostate cancer cell lines.* Prostate, 1993. **23**(2): p. 123-34.

5. Hu, R., et al., *Ligand-independent androgen receptor variants derived from splicing of cryptic exons signify hormone-refractory prostate cancer.* Cancer Res, 2009. **69**(1): p. 16-22.

6. Culig, Z., et al., *Switch from antagonist to agonist of the androgen receptor bicalutamide is associated with prostate tumour progression in a new model system.* Br J Cancer, 1999. **81**(2): p. 242-51.

7. Wu, H.C., et al., *Derivation of androgen-independent human LNCaP prostatic cancer cell sublines: role of bone stromal cells.* Int J Cancer, 1994. **57**(3): p. 406-12.

8. Sramkoski, R.M., et al., *A new human prostate carcinoma cell line, 22Rv1.* In Vitro Cell Dev Biol Anim, 1999. **35**(7): p. 403-9.

9. Dehm, S.M., et al., *Splicing of a novel androgen receptor exon generates a constitutively active androgen receptor that mediates prostate cancer therapy resistance.* Cancer Res, 2008. **68**(13): p. 5469-77.

10. Cunningham, D. and Z. You, *In vitro and in vivo model systems used in prostate cancer research.* J Biol Methods, 2015. **2**(1).

11. Korenchuk, S., et al., *VCaP, a cell-based model system of human prostate cancer.* In Vivo, 2001. **15**(2): p. 163-8.

12. van Bokhoven, A., et al., *Molecular characterization of human prostate carcinoma cell lines.* Prostate, 2003. **57**(3): p. 205-25.

13. Klein, K.A., et al., *Progression of metastatic human prostate cancer to androgen independence in immunodeficient SCID mice.* Nat Med, 1997. **3**(4): p. 402-8.

14. Neshat, M.S., et al., *Enhanced sensitivity of PTEN-deficient tumors to inhibition of FRAP/mTOR.* Proc Natl Acad Sci U S A, 2001. **98**(18): p. 10314-9.

15. Kaighn, M.E., et al., *Establishment and characterization of a human prostatic carcinoma cell line (PC-3).* Invest Urol, 1979. **17**(1): p. 16-23.

16. Koochekpour, S., et al., *Establishment and characterization of a highly tumorigenic African American prostate cancer cell line, E006AA-hT.* Int J Biol Sci, 2014. **10**(8): p. 834-45.

17. Fraser, M., et al., *PTEN deletion in prostate cancer cells does not associate with loss of RAD51 function: implications for radiotherapy and chemotherapy.* Clin Cancer Res, 2012. **18**(4): p. 1015-27.

18. Stone, K.R., et al., *Isolation of a human prostate carcinoma cell line (DU 145).* Int J Cancer, 1978. **21**(3): p. 274-81.
